# Efficacy of probiotics in the prevention of diarrhea in ventilated critically ill ICU patients: meta-analysis of randomized control trials: author’s reply

**DOI:** 10.1186/s40560-021-00578-0

**Published:** 2021-10-15

**Authors:** Priyam Batra, Kapil Dev Soni, Purva Mathur

**Affiliations:** 1 Department of Microbiology, AIIMS, New Delhi, India; 2 Department of Critical and Intensive Care, JPNA Trauma Center, AIIMS, New Delhi, India; 3 Department of Laboratory Medicine, JPNA Trauma Center, AIIMS, New Delhi, India

Sir,

I thank Shimizu et al. for their interest in our paper entitled “Efficacy of probiotics in the prevention of VAP in critically ill ICU patients: an updated systematic review and meta-analysis of randomized control trials” [1]. We are thankful to the authors for their valuable re-analysis of our meta-analysis and bringing forth the additional advantage of diarrhea prevention in ventilated patients by the use of probiotics. We agree that we missed inclusion of their valuable study entitled “Synbiotics modulate gut microbiota and reduce enteritis and ventilator-associated pneumonia in patients with sepsis: a randomized controlled trial” [2] while performing the forest plot analysis of the incidence of diarrhea in ventilated ICU patients. Both the reviewers again analyzed the data and found that as the authors had used the word “enteritis” and “loose stools” in their paper in place of “diarrhea” the study was missed in the analysis.

Most previous meta-analysis such as by Su et al. and Johnstone et al. [3, 4]have missed this important finding. A recently published randomized control study by Johnstone et al. [5] showed that there was no significant difference in diarrhea between patients in the probiotic group vs placebo group. Thus, we re-assessed the meta-analysis after adding this study also to our meta-analysis (as shown in Fig. 1). The effect was seen on a total of 3176 patients (1582 probiotic group vs 1594 placebo group). Moderate heterogeneity (OR 0.63, CI 0.38, 1.04; *P* = 0.07; *I*^2^ = 65%) was seen between the studies. After adding this study, it can be seen that the trend favors probiotics in reducing the risk of diarrhea in ventilated patients though the effect is not statistically significant.

**Fig. 1:**
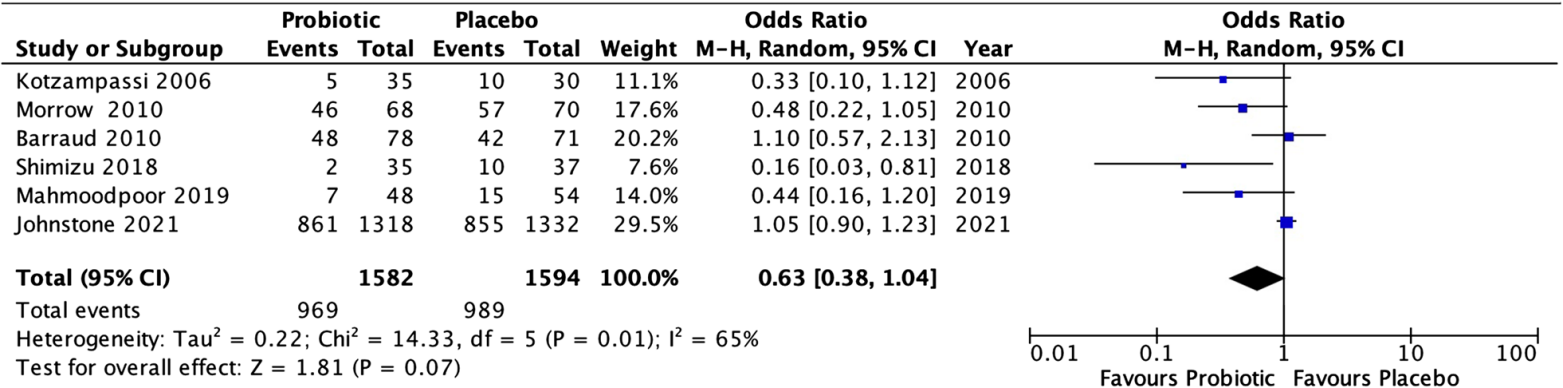
A forest plot of the incidence of diarrhea

The use of probiotics has been well known to be associated with the reduction of C. difficile-associated diarrhea as supported by Goldenberg et al. [6] and Gokalani et al. [7]. This additional knowledge of the role of probiotics in prevention of diarrhea in ventilated patients is valuable and would encourage most clinicians in the use of probiotics in all ventilated ICU patients.

## Data Availability

Available with the corresponding author.
